# The Role of TRP Channels in the Metastatic Cascade

**DOI:** 10.3390/ph11020048

**Published:** 2018-05-17

**Authors:** Benedikt Fels, Etmar Bulk, Zoltán Pethő, Albrecht Schwab

**Affiliations:** Institut für Physiologie II, Robert-Koch-Str. 27b, 48149 Münster, Germany; ebulk@uni-muenster.de (E.B.); pethoe@uni-muenster.de (Z.P.); aschwab@uni-muenster.de (A.S.)

**Keywords:** metastatic cascade, TRP channels, tumor microenvironment, transportome

## Abstract

A dysregulated cellular Ca^2+^ homeostasis is involved in multiple pathologies including cancer. Changes in Ca^2+^ signaling caused by altered fluxes through ion channels and transporters (the transportome) are involved in all steps of the metastatic cascade. Cancer cells thereby “re-program” and “misuse” the cellular transportome to regulate proliferation, apoptosis, metabolism, growth factor signaling, migration and invasion. Cancer cells use their transportome to cope with diverse environmental challenges during the metastatic cascade, like hypoxic, acidic and mechanical cues. Hence, ion channels and transporters are key modulators of cancer progression. This review focuses on the role of transient receptor potential (TRP) channels in the metastatic cascade. After briefly introducing the role of the transportome in cancer, we discuss TRP channel functions in cancer cell migration. We highlight the role of TRP channels in sensing and transmitting cues from the tumor microenvironment and discuss their role in cancer cell invasion. We identify open questions concerning the role of TRP channels in circulating tumor cells and in the processes of intra- and extravasation of tumor cells. We emphasize the importance of TRP channels in different steps of cancer metastasis and propose cancer-specific TRP channel blockade as a therapeutic option in cancer treatment.

## 1. Introduction

Metastasis is the most important factor determining patient prognosis because most cancer patients die from the consequences of metastases [1]. This is particularly relevant for cancers which are usually asymptomatic in early stages and for which no easy screening tools are available such as the determination of prostate-specific antigen (PSA) levels in the plasma for prostate cancer. Thus, pancreatic ductal adenocarcinoma (PDAC) and non-small cell lung cancer (NSCLC) are two examples for cancers that are frequently diagnosed only in an advanced stage when macroscopic metastases have already developed. Accordingly, the prognosis of these cancers is extremely poor with 5-year survival rates of only 5% and 13% for European PDAC [2] and NSCLC patients [3], respectively. Disappointingly, the enormous gain of knowledge with respect to molecular and genetic mechanisms underlying these cancers has not yet led to therapeutic success. Thus, there still remains an unmet need for better understanding the pathophysiology of these diseases. Given the crucial role of metastasis for patient prognosis, it is particularly important to develop new concepts in the understanding of the mechanisms underlying metastasis.

Such a new concept, namely the involvement of ion-conducting transporters in neoplastic diseases has been emerging in recent years. Proteins involved in ion transport (the transportome) were found to contribute to essentially all “hallmarks of cancer” [4]. In this context, it is important to keep in mind that the transportome is the “working horse” of epithelial cells that are the origin of ~90% of all malignant tumors. The transportome is “misused” by cancer cells and regulates, among others, processes such as tumor cell proliferation, apoptosis, senescence, migration, invasion, metabolism and growth factor signaling. This is in part due to the dysregulated expression and function of transportome members in many tumors. Moreover, dysregulated expression frequently correlates with patient prognosis (see [5] for a series of reviews, e.g., [6,7,8,9]). The consistent presence of dysregulated ion channel expression and function in all cancers studied so far [10] has recently led to the provocative question of whether cancer hallmarks can be viewed as oncochannelopathies [11].

In the present review, we will focus on the contribution of transportome members to steps of the so-called metastatic cascade, i.e., on the mechanisms underlying the hematogenous or lymphatic spread of tumor cells from the primary tumor to distant organs. The literature on the cancer transportome has grown exponentially during the last years so that we will limit our discussion primarily to transient receptor potential (TRP) channels (see Table 1). The steps of the metastatic cascade are well defined. After leaving the primary tumor cancer cells invade into and migrate through the neighboring tissue to reach blood or lymph vessels that may have been newly formed (tumor angiogenesis). Following intravasation they are swept away with the bloodstream or the lymph. Within the bloodstream, circulating tumor cells (CTCs) interact with different blood cells such as platelets [12]. Platelet-CTC interactions seem to increase CTC survival, extravasation and metastasis [13,14]. Surviving CTCs adhere to endothelial cells lining the vessels, migrate intravascularly and penetrate the endothelial cell layer (extravasation). Alternatively, individual cancer cells or cell clusters may be trapped mechanically in small capillaries. Finally, the cells expand to form metastases in different parts of the body, and new vessels are formed to supply them with nutrients [1,15]. The extravasation of tumor cells bears many similarities with the recruitment of immune cells [16]. For example, some of the relevant adhesion molecules required for immune cell recruitment, such as LFA-1 or ICAM-1, have also been found in lung adenocarcinoma cells [17,18].

TRP channels are a large family of cation channels all showing sequence homology to the *Drosophila* TRP protein. Its subfamilies present in mammals are: the ankyrin subfamily TRPA, the canonical subfamily TRPC, the melastatin subfamily TRPM, the mucolypin subfamily TRPML, the polycystin subfamily TRPP and the vanilloid subfamily TRPV. They have varying selectivity ranging from nonselective cation channels to highly selective channels (e.g., for Ca^2+^). Their gating is also quite heterogeneous, as they can be gated by e.g., ligands, temperature or mechanical stimuli. [19,20]. TRP channels are involved in a wide variety of cellular processes. Some examples include Ca^2+^ homeostasis, nociception, inflammation, phagocytosis, or cell motility (e.g., reviewed in [21,22,23,24]). Their function can be described in very general terms as that of “cellular sensors”. Thereby, TRP channels confer the ability onto metastasizing cancer cells to respond to ambient physico-chemical signals. Microenvironmental stimuli are of central importance throughout the metastatic cascade. During the metastatic cascade cancer cell behavior is shaped by a wide variety of (harsh) microenvironmental stimuli [1]. Examples of such stimuli along the metastatic cascade and how their potential impact on TRP channel activity can regulate tumor and stromal cell behavior will be the main focus of this review.

There are numerous studies showing a clear correlation between cancer patient survival and TRP channel expression, e.g., TRPC1, TRPM2 and TRPV4 in breast cancer [25,26,27], TRPM7 in PDAC [28], TRPM8 in bladder cancer and osteosarcoma [29,30] and TRPV2 in breast and esophageal cancer [31,32] to name just a few examples (see also Table 2). Since cancer patients usually die from the consequences of metastases, the multitude of these observations strongly indicates that TRP channels have a significant share in the processes underlying the metastatic cascade. Thus, studying the role of TRP channels in steps of the metastatic cascade is a clinically relevant undertaking and bears great therapeutic potential.

## 2. TRP Channels in Cancer Cell Migration and Invasion

Cell migration involves a large variety of temporally and spatially coordinated processes ranging from cell polarization, adhesion/de-adhesion to/from the surrounding matrix and/or neighboring cells, as well as extensive cytoskeletal and membrane dynamics [70,71,72]. Many components of the migration machinery are Ca^2+^-sensitive, including myosin-II [73], focal adhesions [74] or Ca^2+^-sensitive ion channels [72]. Hence, the intracellular Ca^2+^ concentration of migrating tumor (stroma) cells is tightly regulated, both spatially and temporally [75]. In fact, it is widely accepted that ion transport across the plasma membrane via numerous Na^+^, Ca^2+^ and K^+^ channels and transporters is crucial for cell migration [7,72,76]. Additionally, ion and H_2_O fluxes strongly depend on an appropriate plasma membrane potential (V_m_). V_m_ as a key biophysical signal thereby regulates e.g., cell volume and migration and provides the driving force for Ca^2+^ influx. Hyperpolarization and depolarization can directly affect normal cell as well as cancer cell function. A depolarized V_m_ in many cancer types could be linked to e.g., cancer cell proliferation (reviewed in [77]). The following chapter will focus on the role of TRP channels in cancer cell migration and invasion.

Before summarizing recent findings in the field, we would like to critically discuss the experimental approaches. The gold standard is intravital microscopy because cells are migrating in their complex physiological environment. But it is not trivial to control and manipulate individual components of the tumor microenvironment such as pH or mechanical properties. Controlling the ambient conditions is more easily achieved in an in vitro setting, yet in a reductionist fashion. Boyden chambers for example are popular for in vitro assays of tumor cell invasion. A frequently used protocol is to coat the filter membranes with matrix proteins and induce “invasion” by applying a chemotactic gradient. However, under such experimental conditions it is impossible to distinguish “invasion” from chemotaxis. Inhibiting chemotaxis will lead to the same readout as inhibiting “invasion” since the steering mechanisms can be affected without impairing the migration motor [78,79]. Inhibiting either mechanism will lead to a reduced number of cells reaching the lower compartment of the Boyden chamber. Unfortunately, the proper control experiments, i.e., the use of Boyden chambers in the absence of chemotactic gradients are not always performed. Wound healing assays are also often used for migration analysis and can be a suitable approach to evaluate directionality of cell movement. Migration analysis within a 3D matrix as well can be a useful tool to analyze the matrix invasion of tumor cells; here, translocation as well as matrix digestion via e.g., MMP secretion should be taken into account. The papers cited below are mainly limited to studies considering the above-mentioned criteria.

### 2.1. TRPM7

TRPM7 is involved in several aspects of cell motility such as polarization [80], adhesion [81,82] and migration [83]. It is a Ca^2+^- and Mg^2+^-permeable channel with an α-kinase domain [84]. TRPM7 is essential for PDAC progression and invasion. TRPM7 expression in primary tumors is associated with PDAC lymph node metastasis [28]. Its over-expression correlates with increased tumor size and advanced tumor stages of pancreatic cancer and hence, inversely with patient prognosis [47,85]. Silencing of TRPM7 in PDAC cell lines leads to a reduction of cancer cell invasion [28]. TRPM7 function in PDAC invasion can be partly explained by TRMP7-mediated Mg^2+^ influx and subsequent kinase activation and heat shock protein secretion. Activation of TRPM7 and Hsp90a secretion could be linked to MMP-2 secretion which is an important step to degrade surrounding ECM and initiate cancer cell invasion [28]. In the non-small cell lung cancer (NSCLC) cell line A549 TRPM7 is upregulated after epidermal growth factor (EGF) stimulation and contributes to EGF-mediated increase in cell migration. Accordingly, shRNA-based silencing of TRPM7 attenuates the stimulation by EGF [48]. In breast cancer, myosin-II-based cell tensions and de-adhesion of cell-matrix contacts are TRPM7-dependent and TRPM7 is necessary for breast cancer metastasis into the lung in a murine model [49] (see also below). Silencing of TRPM7 in MDA-MB-435 breast cancer cells leads to a reduced migration. This can be accounted for a TRPM7-dependent regulation of Src and MAPK kinase pathways [51]. However, it should be noted that the origin of MDA-MB-435 cells has recently been questioned [65]. These findings suggest that at least part of the role of TRPM7 is calcium-independent. It rather involves its α-kinase domain which is needed for phosphorylation of myosin-IIA heavy chain [50,51,86]. A similar role of TRPM7 in cancer cell migration was found in a number of other tumors such as nasopharyngeal carcinoma and ovarian cancer [53,54]. In addition, TRPM7 is also known for its role in tumor cell proliferation [65,87,88].

### 2.2. TRPM8

There are numerous reports describing a role for TRPM8 in cancer cell migration and invasion. However, depending on the type of cancer its impact may be pro- or anti-migratory. In oral squamous cell carcinoma, activation of TRPM8 leads to an increase of MMP-9 activity and cell migration [55]. In breast cancer, TRPM8 promotes the aggressiveness of breast cancer cells by regulating epithelial-mesenchymal transition (EMT) via the activation of the AKT/GSK-3β pathway [56]. Silencing of TRPM8 decreases and over-expression increases the motility of MDA-MB-231 or MCF-7 breast cancer cells. Activation of TRPM8 in combination with TRPA1 leads to enhanced motility also in lung cancer cells, whereas knockdown shows the opposite effect [33]. In glioblastoma cells, TRPM8 inhibition reduces the migration and chemotaxis [57]. In contrast, in PDAC cells and in prostate cancer cells TRPM8 function and expression reduces cell motility [58,59,89]. TRPM8 function is also linked to cell survival in prostate cancer and TRMP8 suppression lead to oxidative stress and apoptosis [60]. TRPM8 channels are also regulated by intracellular proteins, so called TRP channel-associated factors (TCAFs). E.g.,TCAF1 leads to a reduction of prostate cancer cell velocity and migration directionality [90]. A modification of TRPM8 channels can also be mediated via N-glycosylation in HEK293 cells, but not in PDAC cell lines Panc-1, MiaPaCa2 or BxPc3, which express non-glycosylated channel isoforms [91]. Independently of its conductive function, TRPM8 acts as a Rap1 GTPase inhibitor, inhibiting endothelial cell migration [92].

### 2.3. TRPV2

Some TRPV channels have been found to be involved in cell proliferation, apoptosis, angiogenesis, migration, invasion, and generally in cancer progression [93]. The elevated expression of TRPV2 in metastatic prostate cancer points to a role of this channel in the metastatic cascade [61]. TRPV2 promotes cell migration and the invasive cancer cell phenotype [94]. Antimicrobial peptide LL-37, which is released from infiltrating immune cells, is able to activate TRPV2 and the Ca^2+^-activated K^+^ channel (K_Ca_1.1) in different breast cancer cell lines leading to increased migration [62]. High TRPV2 expression is also correlated with a poor prognosis of esophageal squamous cell carcinoma (ESCC) [32].

### 2.4. TRPV4

TRPV4 is upregulated in breast cancer patients. TRPV4-mediated activation of protein kinase B leads to enhanced Akt and focal adhesion kinase (FAK) phosphorylation, which are both associated with cell migration. Pharmacological activation of TRPV4 in a breast cancer cell line causes downregulation of adhesion molecules like E-cadherin and β-catenin [26]. Upregulation of TRPV4 is accompanied by changes of the cytoskeletal network, enhanced blebability and reduced cell stiffness, facilitating cancer metastasis through neighboring tissues. It was suggested that TRPV4 activation thereby regulates breast cancer cell extravasation [63]. In gastric cancer, TRPV4 can be activated by co-localized calcium-sensing receptors, leading to Ca^2+^-induced proliferation, migration and invasion [64]. TRPV4 channels are also important players in tumor vascularization by regulating endothelial cell migration [95] (see chapter “TRP channels in tumor vascularization”). Moreover, they modulate the endothelial barrier permeability (see chapter “Extravasation of tumor cells” below).

### 2.5. TRPV6

TRPV6 is associated with both pancreatic and breast cancer. TRPV6 is upregulated in human pancreatic cancer specimens and silencing of TRPV6 significantly inhibits invasion, proliferation and migration of pancreatic cancer cells [66]. In breast cancer, TRPV6 expression is higher in invasive areas of breast cancer tissues in comparison to the corresponding non-invasive areas. Moreover, TRPV6 silencing inhibits MDA-MB-231 and MCF-7 breast cancer cell migration. Therefore, TRPV6 is suggested to be involved in the metastatic process of breast cancer [65].

### 2.6. TRPC1

TRPC1 is required for PDGF- and EGF-mediated directional migration in glioma cells [39,96] by cooperating with chloride channels that are activated by TRPC1-mediated Ca^2+^ influx [97]. TRPC1 channels are also necessary for VEGF signaling and thyroid cancer migration and invasion [40]. TRPC1-mediated Ca^2+^ influx is needed for NSCLC proliferation in response to EGF signaling [37]. Transforming growth factor β1 (TGF-β1) induces Ca^2+^ entry likely via TRPC1 and NCX1 that raise cytosolic Ca^2+^ in pancreatic cancer cells so that knockdown of TRPC1 reverses TGF-β1-induced pancreatic cancer cell motility [36]. Silencing of TRPC1 or its activator STIM1 reduce TGF-β1 mediated calpain activation and subsequent cell migration, as well as expression of EMT markers such as N-cadherin and vimentin [34]. TRPC1 together with STIM1/Orai1 is also needed for colon cancer cell line (HCT-116) migration [98].

### 2.7. TRPC4

TRPC4 contributes to enhanced invasion and metastasis of granule precursor-derived human medulloblastoma [41]. Here, the authors use OGR1 to induce the expression of TRPC4 which promotes the migration of medulloblastoma cells.

### 2.8. TRPC5

It has been shown that overexpression of TRPC5 correlates with a poor prognosis in colon cancer by promoting tumor metastasis via the hypoxia-induced factor 1α (HIF-1α) -Twist signaling pathway [42].

### 2.9. TRPC6

TRPC6 is linked to several cancer types such as prostate, lung and colon cancer as well as glioblastoma. In prostate cancer, TRPC6 is suggested to be involved in cancer cell invasion into a “matrigel-based” matrix [99]. TRPC6 is detected in benign and malignant human prostate tumor tissues as well as in prostate cancer cell lines and its expression levels are associated with the histological grade [45]. Expression profiles of some TRP channels including TRPC6 are changing during the progression of prostate cancer towards the more aggressive and hormone-refractory stages [100]. TRPC6 also has been linked to lung cancer [44]: inhibition of TRPC6 channels lowers the intracellular Ca^2+^ concentration in A549 cells and strongly reduces the invasion of A549 cells. In human glioblastoma cells, TRPC6 expression is augmented by hypoxia and increases proliferation and cell invasion [43]. Here, TRPC6 is suggested to be a key mediator of Notch-driven glioblastoma invasiveness and angiogenesis. TRPC6 was also found to be upregulated in esophageal squamous cell carcinoma in which it negatively correlates with patients survival [68].

### 2.10. Other TRP Channels

The TRPA1 channel is involved in cellular invasion. It has been shown with transwell-invasion-assays that methyl syringate, a TRPA1 agonist inhibits induction of COX-2 and cell invasion of the NSCLC cell line A549 and of the fibrosarcoma cell line HT-1080 under hypoxic conditions [101].

To the best knowledge of the authors, there is no direct evidence linking TRPML channels to cancer invasiveness and metastasis, although the channel has an altered expression in glioblastoma and breast cancer [102]. This might be due to the subcellular localization of TRPML channels, as they are primarily expressed in endosomes and lysosomes, and they are not as easily investigated as channels residing in the plasma membrane. Moreover, the literature focuses on the effect of TRPML on autophagy and autophagy-related signaling that is independent of the calcium permeability of the channel [102]. However, these channels are key regulators of lysosomal calcium release, and moreover, their expression is linked to ERK1/2 and Akt signaling which plays a role in cell migration.

## 3. Influence of the Tumor Microenvironment on TRP Channels

The initial step of cancer cell invasion and migration out of the primary tumor is strongly regulated by a variety of tumor microenvironmental factors like pH, hypoxia, matrix stiffness, cytokines and the infiltrating cellular components such as fibroblasts and immune cells. The following chapter will link these factors to TRP channel function in tumor progression and invasion.

3.1. pH

Due to insufficient vascularization, limited supply of metabolic substrates, metabolic reprogramming towards the so-called aerobic glycolysis (Warburg effect) and-at least in later stages–tumor anemia, primary tumors and cancer cells export increased amounts of H^+^ and the tumor microenvironment (TME) becomes acidified [103,104,105,106]. In PDAC the extracellular pH landscape is superimposed by an intermittent postprandial acidification of the interstitium which is a consequence of the massive HCO_3_^-^ secretion into the pancreatic ducts [107,108]. Similarly, the pH landscapes of stomach, bone or skin tumors are superimposed by the respective characteristic acid-base homeostasis of these organs. Several TRP channels are regulated by an extra-/intracellular acidosis [19,109]. They can either be activated, e.g., TRPV1/3 [20], TRPC5 [110] or inhibited by an acidosis such as TRPM2 [111] and TRPM8 [112], or their selectivity is regulated by pH (e.g., TRPM7; [113]). There is a close interrelation between the regulation of TRPM7 by protons and the divalent cations Ca^2+^ and Mg^2+^. On the one hand, the effect of the extracellular pH on TRPM7 activity depends on the presence of extracellular Ca^2+^ and Mg^2+^ [114]. On the other hand, the selectivity of TRPM7 channels for mono- or divalent cations is regulated by the extracellular pH. An acidification (e.g., pH 6) increases the permeability for monovalent cations [113]. For tumor cells, the impact of an intracellular alkalization (pH >7.2) is more relevant because tumor cells are frequently characterized by an alkaline intracellular pH [105]. TRMP7 channels are activated by an alkaline pH in these cells [115].

TRPV1 is a nonselective cation channel that is not only regulated by protons but also permeable for protons [20,116]. Low extracellular pH in lymphatic endothelial cells induces lymph-angiogenesis in a TRPV1-dependent manner [117]. This pH-dependent activation of TRPV1 also induces cytokine production (IL8) by endothelial cells.

The effect of pH on TRPV4 cells is controversially. Heterologously expressed TRPV4 channels can be activated by a drop of extracellular pH in CHO cells [118]. However, in esophageal epithelial cells TRPV4 activity is suppressed by an acidic pH [119].

TRPC4 and TRPC5 channels are already activated by a modest decrease of the extracellular pH (e.g., pH 7.0) when expressed in HEK293 cells [110].

TRPM2 channels are inhibited by extracellular acidification (pH 6.5) as protons compete with other cations like Ca^2+^ or Na^+^ for channel permeation and act as competitive TRPM2 antagonists [111]. Another possible mechanism for proton-dependent regulation of TRP channel activity was shown for TRPM8; the channel activity is reduced by extracellular divalent cations as well as by protons. The authors showed that this is due to a change in the membrane surface charge caused by an increased proton concentration [112].

### 3.2. Hypoxia

The high metabolic demand of the tumor cells and the insufficient blood and oxygen supply due the compression and inadequate number of vessels frequently cause hypoxia of the tumor stroma [120,121,122]. Hypoxia can be sensed by several TRP channels such as TRPM7 and TRPA1 [123], TRPC1 [25,40], TRPC6 [43,124,125,126], both in cancer cells and in stromal cells [126,127]. Hypoxia is commonly associated with increased production of reactive oxygen species (ROS) that can elicit both protumorigenic and antitumor effects [128]. TRPC6, TRPV1, TRPM2/4/7, TRPA1 are activated by ROS [19], and they are regulators of relevant steps of the metastatic cascade.

TRPM2 channels can be activated by hypoxia. They are expressed in several cancer types, for instance in breast cancer [129], neuroblastoma [130] and malignant melanoma [131]. In neuroblastoma cells, TRPM2 depletion leads to a suppressed HIF-1α signaling. Inhibiting TRPM2 function by mutating its pore region increases mitochondrial ROS production [132]. Accordingly, TRPM2 activation by oxidative stress in ischemic hearts prevents ROS production in the hypoxic heart [133]. A protective role of TRPM2 under hypoxia can also be found in neuroblastoma cells, in which activated TRPM2 channels lead to increased expression of superoxide dismutase 2 (SOD2) and reduced ROS levels [130,132].

TRPM7 as well as TRPA1 can be activated by hypoxia and increased ROS levels [123,134,135]. The prominent role of TRPM7 channels in cancer progression and cancer cell migration has already been discussed (see “TRP channels in cancer cell migration and invasion”).

TPRV1 and TRPV4 channels are activated under hypoxic conditions in pulmonary artery smooth muscle cells and induce migration [136].

In the follicular thyroid cancer cell line ML-1, knockdown of TRPC1 channels leads to decreased HIF-1α levels [40]. Corresponding results were found in breast cancer cells, in which hypoxia increases TRPC1 expression and TRPC1 regulates hypoxia-induced signaling [25].

In glioma cells, TRPC6 is upregulated and required for the hypoxia-mediated increase in proliferation and cell invasion [43]. Activated TRPC6 stabilizes HIF-1α in hypoxic glioma cells and supports hypoxic glucose metabolism of cancer cells via the GLUT1 transporter [124]. TRPC6 channels are also activated in a hypoxic environment in pancreatic stellate cells, which are the main producers of extracellular matrix proteins in PDAC [126,137]. Similar results were found in hepatic stellate cells, in which TRPC6 expression increases under hypoxic conditions [127].

### 3.3. Cytokines

The tumor microenvironment is a rich source of cytokines and growth factors secreted by tumor cells, stromal cells and infiltrating immune cells. Cytokine and growth factor receptors frequently trigger TRP channel-dependent signaling cascades [137]. In addition, TRP channels can also modify cytokine secretion of cancer and stroma cells and thereby modify the composition of the tumor microenvironment. However, it must be stated at this point that many of the studies investigating the role of TRP channels in cytokine secretion were not made with tumor cells. Nonetheless, they can be taken as proof-of-principle and studies made with immune cells are highly relevant because of the consistent immune cell infiltration of the tumor stroma.

TRPM2 is important for secretion of IL-2, IFNү and IL-17 in T-lymphocytes [138] as well as for IL-8 secretion in monocytes [139]. Stimulation of TRPM3 channels leads to activation of extracellular signal-regulated protein kinase (ERK1/2) and transcription of IL-8, a proinflammatory CXC chemokine important for proliferation, survival and migration of cancer cells in lung cancer and PDAC [140,141,142,143]. TRPM8 inhibition in murine peritoneal macrophages leads to a pro-inflammatory cytokine profile with increased TNFα and decreased IL-6 secretion [144]. TRPM8 activators like eucalyptol inhibit secretion of TNFα, IL-1β, IL-6 and IL-8 in monocytes [145]. In lung epithelial cells, TRPM8 activation increases transcription of IL-1α, IL-1β, IL-4, IL-6, IL-8, IL-13 and TNFα [146].

Ca^2+^ entry through TRPV2 was shown to be involved in IL-6 secretion in RAW264 and murine macrophages [147]. IL-8 secretion in human non-melanoma skin cancer can be stimulated by TRPV4, thereby leading to a possibly inhibitory autocrine circuitry, because IL-8 induces the downregulation of TRPV4 channels [148]. In human airway epithelial NCI-H292 cells, TRPV4 activation triggers Ca^2+^ entry and release of IL-8 and prostaglandin E_2_ (PGE_2_) in vitro and increases KC levels in in vivo murine bronchoalveolar lavage fluids [149].

### 3.4. Mechanical Properties of Tumor Cells and the Tumor Microenvironment

In addition to the above mentioned chemical stimuli, metastasizing tumor cells and stromal cells are also exposed to mechanical stimuli [150]. Altered mechanics is one of the main characteristics of most cancer types. In fact, the clinical detection of a tumor by palpation relies on the typical mechanical properties of the tumor tissue [150,151]. The elasticity of the tumor stroma [150,152] and the tissue pressure [121,153] are usually higher than those of the normal organs. PDAC and breast cancer may serve as prominent examples [154,155,156,157]. Altered mechanical properties not only stimulate the cancer cells. They have an equal impact on stromal cells as illustrated by the response of pancreatic stellate cells to a mechanical load. They are activated by an elevated tissue pressure and substrate rigidity [152,158].

Moreover, circulating tumor cells are exposed to massive shear forces while in the bloodstream and prior to extravasation. Finally, tumor and stroma cell migration itself also generates mechanical signals within the cells that–via TRP channel activation–modulate the migratory behavior [159]. There is ample evidence that TRP channels are involved in mechano-signaling [125]. TRPC1 [158,160], TRPM7 [161], TRPV4 [63], TRPA1 channels [162] are some of the relevant TRP channels that are also required for specific steps of the metastatic cascade.

TRPC1 channels are found at the rear end of polarized U2OS osteosarcoma cells. TRPC1 knockdown leads to disturbed cell polarity, decreased cell stiffness and disorganization of the actin filaments and microtubules [163]. In pancreatic stellate cells TRPC1 is involved in responding to an increase of the ambient pressure and its activation leads to increased migratory activity of pancreatic stellate cells [158]. TRPC1 also contributes to mechano-signaling during migration of MDCK-F cells and fibroblasts. Knockdown or knockout of TRPC1 attenuates calcium transients following mechanical stretch. Moreover, TRPC1 channels are needed for MDCK-F cells to respond to directional mechanical cues [160].

TRPM7 can be activated by mechanical stimuli like membrane stretch as well as through phospholipase C (PLC) signaling [164,165]. TRPM7 channels are involved in calpain signaling and myosin-II activation and modulate actomyosin cytoskeleton contraction [81,86]. In neuroblastoma cells TRPM7 modulates the cytoskeletal organization and affects the malignancy of tumor cells by regulating actomyosin dynamics and cell-matrix interactions [166].

In tumor-derived endothelial cells TRPV4 expression levels are lowered. This leads to decreased mechanosensitivity and increased cell spreading on stiff matrices—an effect that is restored by overexpression of TRPV4. Prostate cancer-derived endothelial cells with low TRPV4 expression showed increased migration and abnormal angiogenesis [167]. TRPV4 is also required for breast cancer cell invasion. In breast cancer, TRPV4 overexpression leads to cancer cell softening, increased cell blebbing and actin reorganization. Altered mechanics were assessed indirectly with a micropipette aspiration technique. This was proposed to point to a role of TRPV4 channels in cancer cell extravasation by reducing cancer cell rigidity and improving the ability of cancer cells to infiltrate through the surrounding tissue [63].

### 3.5. Stroma Cells

The function of stromal cells such as fibroblast and immune cells, that are recruited and activated by cancer cells, is regulated by different TRP channels as well [137]. Our group has begun a systematic analysis of the (TRP) channels regulating the function of pancreatic stellate cells. These cells play a major role in PDAC progression by underlying the desmoplastic reaction within the tumor stroma and they are also involved in acute and chronic pancreatitis. Their functions rely on intracellular Ca^2+^ signaling [168,169]. Blockade of Ca^2+^-release-activated Ca^2+^-channels (CRAC) by GSK-7975A inhibits Ca^2+^ signaling in pancreatic stellate cells and attenuates pancreatitis [169].

Activation and migration of pancreatic stellate cells depend on TRPC1 channel expression. As mentioned in the preceding paragraph, TRPC1 is involved in the mechano-signaling of murine pancreatic stellate cells and needed for pressure-dependent activation [158]. TRPC3 channels are upregulated in the PDAC stroma. By cooperation with K_Ca_3.1 channels they are necessary for pancreatic stellate cell migration and chemotaxis by mediating Ca^2+^ influx necessary for calpain stimulation [170]. TRPC6 channels are required for hypoxia-induced activation and autocrine stimulation of pancreatic stellate cells [126].

In hepatic stellate cells, TRPM7 is in involved in cell activation via the ERK and phosphoinositide 3-kinase (PI3K) pathway [171]. Inhibition or knockdown of TRPM7 lead to reduced proliferation and attenuated expression of activation markers like α-smooth muscle actin and Col1α1 in response to platelet-derived growth factor (PDGF) and TGF-β1 stimulation [171,172]. TRPM7 also underlies Ca^2+^ signaling at the leading edge of migrating lung fibroblasts [173]. In prostate cancer-associated fibroblasts, activation of TRPA1 leads to increased Ca^2+^ levels and elevated hepatocyte growth factor (HGF) and vascular endothelial growth factor (VEGF) secretion. Co-cultured prostate cancer cells are rescued from apoptosis by TRPA1 activation [174].

Infiltrating immune cells like neutrophils are important constituents of the tumor stroma in numerous tumors, including PDAC [175,176]. Neutrophils are recruited to the PDAC stroma via CXCR2 signaling [177] which in turn relies on TRPC6 channel activity [79]. It is discussed controversially whether TRPM2 channels contribute to neutrophil chemotaxis [139,178,179,180] Other neutrophilic TRP channels that are linked to chemotaxis or tissue infiltration include TRPM7 [139,178,179,180] and TRPV4 [181]. For a detailed overview of TRP channel function in neutrophil granulocytes we refer to a recent review from our group [23].

Taken together, the tumor microenvironment encompasses a wealth of different stimuli that shape cancer and stromal cell behavior. TRP channels are not only the sensors for these stimuli, they are also involved in transducing these external stimuli to altered cellular behavior and finally modify the tumor microenvironment by inducing e.g., cytokine secretion.

## 4. TRP Channels in Tumor Vascularization

Tumor cells must have access to blood or lymph vessels in order to spread within the body. They can either enter already existing vessels in the host organ, or they can enter newly formed vessels within the tumor. Thus, tumor angiogenesis is generally a prerequisite for metastasis. Tumor angiogenesis is initiated among others by growth factors secreted by tumor cells into the hypoxic tumor microenvironment such as VEGF, EGF and many others [95]. It strongly relies on intracellular Ca^2+^ signaling which may in part be mediated by TRP channels (reviewed in [95,182,183]). However, there are discrepancies between in vitro and in vivo studies. While the former reveal a participation of TRPC1 and TRPC6 channels in endothelial tube formation [184,185], the respective knockout mice appear to have a normal vasculature [3,186]. On the other hand, modulating TRPV4 channel activity pharmacologically (4α-PDD; [187]) or mechanically [188] elicits concordant effects in vivo and in vitro. It is interesting to note that altered expression of TRP channels is also observed in tumor-derived endothelial cells. This has been well documented for TRPV4 channels in breast cancer and prostate cancer-derived endothelial cells [167,189,190].

## 5. Extravasation of Tumor Cells

Extravasation of circulating tumor cells may originate from single tumor cells or cell clusters [12]. Prior to crossing the vessel wall, tumor cells have to adhere to endothelial cells. For extravasation of single tumor cells, analogies with immune cells are evident. Accordingly, adhesion molecules expressed by endothelial and tumor cells are crucial. Prominent examples are the family of cadherins, selectins, integrins or the Ig superfamily, including ICAM-1 or VCAM-1. There are several examples showing that the expression of adhesion molecules in endothelial cells is modulated by Ca^2+^-permeable ion channels. Overexpression of Orai1 potentiates the expression of ICAM-1 and VCAM-1 [191], silencing of TRPC1 attenuates cisplatin-induced ICAM-1 expression and endothelial dysfunction [192] and overexpression of TRPC3 enhances TNFα-induced VCAM-1 expression. Binding of selectins to their ligands is Ca^2+^-dependent. P-selectin has been identified to mediate adhesion of various leukocytes and certain types of cancer cells [193]. Its expression in endothelial cells is upregulated by TRPV4 agonists [194]. E-selectin with its ligands sialyl-Lewis-a or sialyl-Lewis-x and CD44 is important for the early attachment of cancer cells to endothelial cells [15]. Most studies only investigate the invasion of tumor cells which precedes intravasation and follows extravasation. We have referred to the role of TRP channels therein in one of the earlier sections of this review.

By analogy with the increased adhesion of monocytes to endothelial cells that is indirectly mediated by TRPC3 channels [195], one would expect a similar behavior for tumor cells as well. Channel-mediated upregulation of endothelial vascular cell adhesion molecule (VCAM)-1 should lead to an increase of adhesion of tumor cells to the endothelium. However, so far there are only very few studies investigating this aspect of the metastatic cascade. Using single cell force spectroscopy we recently measured cell-cell adhesion forces between A549 non-small cell lung cancer cells and human microvascular endothelial (HMEC-1) cells. We could show that inhibition or silencing of the Ca^2+^-activated potassium channel K_Ca_3.1, which, by transporting positive charges to the extracellular space, provides electrical driving force for Ca^2+^ influx via TRP channels [170], increases ICAM-1-dependent adhesion between A549 and HMEC-1 cells [17]. One important conclusion of our study is that the adhesion of tumor cells to endothelial cells is largely regulated by *endothelial* K_Ca_3.1 channels. Since inhibition of K_Ca_3.1 channels leads to a decrease of the intracellular Ca^2+^ concentration [170,196], these channels must regulate ICAM-1 expression in a different manner than Orai1 or TRPC1 which mediate an increase of the intracellular Ca^2+^ concentration [184,185].

The limited knowledge on the role of ion channels in cell-cell adhesion contrasts with that on cell-matrix adhesion. Several studies have shown a role of TRP channels in this process. A few examples are listed in the following: inhibition of TRPC1 decreases adhesiveness of CNE2 nasopharyngeal tumor cells [38], TRPC2 channels regulate adhesion of rat thyroid FRTL-5 cells [197], silencing TRPM7 channels increases the adhesiveness of human umbilical vein endothelial (HUVEC) cells [82] and TRPM8 activation leads to inhibition of the GTPase Rap1 and impaired ß1 integrin-dependent adhesion and migration of endothelial cell line (HMECs) [92].

Once tumor cells are adherent to endothelial cells they will eventually breach the endothelial barrier and invade the underlying tissue. It is well known that cadherin-mediated cell-cell adhesion of endothelial cells is Ca^2+^-dependent [198]. The cadherin-mediated barrier integrity also depends on intracellular Ca^2+^ signaling that in turn is regulated, among others, by TRPV4 channels as shown for retinal endothelial cells [199] or for pulmonary vessels [12,200]. Endothelial TRPV4 channels cooperate with K_Ca_3.1 channels in the regulation of the endothelial barrier integrity [201]. The barrier integrity can also be modulated by endothelial TRPM2 channels. When they are activated by oxidants generated by neutrophil granulocytes, endothelial cell junctions open and facilitate transmigration of neutrophils [202]. It remains to be seen whether such a mechanism also applies for tumor cell extravasation. Moreover, endothelial TRPC6 [203] and K_2P_2.1 channels [204] control the transendothelial migration of leukocytes. To the best of our knowledge, a role of TRP channels in transendothelial migration of tumor cells has not been directly shown. Our study showing that inhibition of endothelial K_Ca_3.1 channels also impairs lung cancer cell transmigration [17] may serve as a further proof-of-principle for the role of ion channels in this process.

## 6. Pharmacologic Targeting of TRP Channels in Cancer

This review emphasizes the therapeutic potential of targeting TRP channels in cancer. TRP channels can already be used as prognostic and predictive clinical markers because TRP channel expression strongly correlates with patient survival (e.g., [205,206,207,208,209]). The pharmacological targeting of TRP channels offers the advantage that tumor, stroma and immune cells can be targeted simultaneously with potentially only one drug. For example, TRPC6 channels are expressed in hepatocellular carcinoma cells [210], in hepatic stellate cells [127], endothelial cells [203] and in neutrophil granulocytes [79]. Moreover, their inhibition is at least partially effective in tumors that are resistantt to chemotherapy [210]. Thus, TRP channel blockade may not only interrupt the mutual activation of tumor and stroma cells. When combined with conventional chemotherapeutics it also offers the opportunity to reduce their dosage and thereby lessen the severity of side effects. The challenge will be to selectively target TRP channel modulators to the tumor in order to avoid systemic side effects. We refer to the review by Gautier and colleagues for an overview of the pharmacological approach of targeting TRP channels in cancer [211].

Some approaches use the fact that TRP channels are upregulated in tumorigenic cells, so that targeted TRP channel activation leads to Ca^2+^ and Na^+^ influx, disruption of the ionic homeostasis and subsequent cell death. For the TRPM8 activator D3263, a clinical Phase 1 dose escalation study (NCT00839631) led to disease stabilization in prostate cancer patients. Another TRPM8 activator, WS-12, may be used as a diagnostic marker for prostate cancer by incorporating radiohalogens [212,213]. Another example is the Phase 1 trial of SOR-C13 (NCT01578564), a TRPV6 inhibitor. TRPV6 inhibition aims to suppress Ca^2+^-mediated cancer proliferation and metastasis e.g., in SCLC, prostate or pancreas cancer [66,214,215].

For ion channel inhibitors, the possible target is typically located in plasma membrane. The drug must only diffuse through the capillaries and the tissue to reach its target. Disease- and drug-derived factors (e.g., interstitial pH) significantly contribute to the differential distribution in the tissues. Therefore, relying solely on total drug concentration has the potential to introduce significant errors into the interpretation of drug delivery mechanisms. To assess the pharmacokinetic properties of potential drugs, it is crucial to also assess the free, unbound drug concentration, which refers to compounds that are for example not bound to plasma proteins [216]. In case of TRP channel inhibitors, it is still necessary to assess the amount of unbound drug concentration in the vicinity of the migrating cancer cells.

However, most of the cancer-associated TRP channels are far away from being targeted in clinical trials and developing suitable TRP channel modulators in combination with cancer-specific applications is urgently needed.

## 7. Conclusions and Open Questions

**Figure 1:**
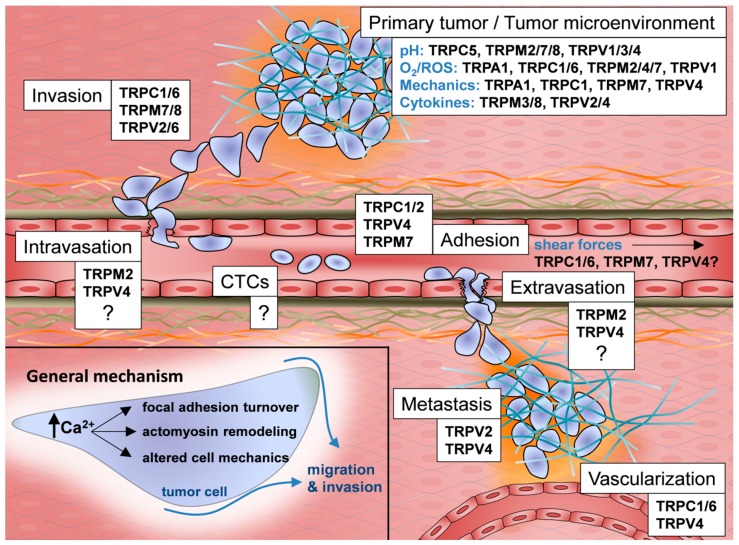
TRP channels in the metastatic cascade.

Recapitulating our knowledge on TRP channel regulation and function, it becomes obvious, that they play an important role in the dissemination of cancer cells and in disease progression (see Figure 1). In our view their contribution to cancer progression can be attributed at least in part to the fact that they are multimodal sensors and transducers/effectors of microenvironmental cues encountered by cancer cells during the metastatic cascade. They respond to many of the chemico-physical stimuli that are relevant for the metastatic spread of cancer cells. In light of the prognostic relevance of metastases, it is not surprising that there are strong correlations between TRP channel expression in tumors and the overall survival of the diseased patients. This review also shows that TRP channel functions in metastasis and cancer in general are still far from being fully understood. Many studies do not provide a detailed look on the mechanistic steps and signaling cascades regulated by TRP channels. Moreover, there is an almost complete lack of knowledge concerning the role of TRP channels in the processes of intra- and extravasation. It is equally unknown whether they play a role in circulating tumor cells. Circulating tumor cells are exposed to a massive mechanical stress so that one can expect a strong activation of mechanosensitive TRP and PIEZO channels potentially leading to a Ca^2+^ overload. While the overexpression of many TRP channels involved in mechano-signaling promotes aggressive tumor cell behavior, this may not be the case for the “success” of circulating tumor cells.

During the metastatic cascade, cancer cells have to respond to various chemico-physical stimuli such as changes in pH, hypoxia, ROS, mechanical cues and growth factor and cytokine gradients. Cancer cells use TRP channels to sense, modify and regulate these stimuli during the different steps of the metastatic cascade/during tumor progression. This figure highlights important TRP channels within the metastatic cascade. Open questions are stressed with question marks.

There are clearly two major quests for the field of TRP channels in cancer. One is to delineate in more detail the contribution of TRP channels to the metastatic cascade. A molecular understanding of the role of TRP channels therein is a prerequisite for developing new (TRP channel-targeting) therapeutic concepts. Novel microfluidic techniques as well as biophysical techniques such as atomic force microscopy will be valuable approaches for deciphering the role of TRP channels in intra- and extravasation of tumor cells and also in circulating tumor cells. The second challenge is to continue the development of new and specific TRP channel modulators so that the therapeutic potential of aberrant TRP channel function in the metastatic cascade can be evaluated pharmacologically and eventually be translated clinically. In our view, the responsiveness of TRP channels to cues from the tumor microenvironment makes them very attractive targets. Their attractiveness is reinforced by the fact that they allow simultaneous targeting of both tumor and stromal cells. Moreover, there is growing evidence that TRP channel modulation constitutes a new approach to overcome resistance to “conventional” cancer therapeutics (e.g., [210,217]). Thereby, the vicious cycle of mutual activation of tumor and stromal cells can potentially be interrupted.

## Figures and Tables

**Table 1:** TRP channels and their impact in malignant phenotypes.

Channel	Cancer Type	Cell line/Tissue	Function/Phenotype	Ref.
TRPA1	Lewis Lung carcinoma	LLC-2	adhesion	[33]
TRPC1	Breast cancer	MDA-MB-231/MCF-10A	migration proliferation	[34] [35]
	Pancreatic cancer PDAC	BxPc3 Capan-1	migration	[36]
	Non-small cell lung cancer (NSCLC)	A549/H1299	proliferation	[37]
	Nasopharyngeal carcinoma	CNE2	adhesion	[38]
	Glioblastoma	U251	migration	[39]
	Thyroid cancer	ML-1	migration, invasion	[40]
TRPC4	Medulloblastoma	DAOY, ONS76, UW228-1	migration	[41]
TRPC5	Colon cancer	SW620/HT29/tissue	proliferation, migration, invasion	[42]
TRPC6	Glioblastoma	U373MG	tumor growth, invasion, angiogenesis	[43]
	NSCLC	A549	proliferation, invasion	[44]
	Prostate cancer	tissue	expression in metastatic tissues	[45]
	Liver cancer	Huh-7/tissue	proliferation, expression	[46]
TRPM7	Pancreatic cancer PDAC	Panc-1/MiaPaCa2/tissue	proliferation, invasion	[28,47]
	NSCLC	A549	migration	[48]
	Breast cancer	MDA-MB-231/tissue MDA-MB-231 MDA-MB-435 * MDA-MB-468	migration, adhesion, cell tension, lung metastasis migration, expression in invasive ER^-^ ductal carcinoma tissue migration, EMT transition	[49] [50] [51] [52]
	Nasopharyngeal carcinoma	NPC SUNE1/5-8F	migration	[53]
	Ovarian cancer	SKVO-3	migration, adhesion, colony formation	[54]
TRPM8	Oral squamous cell carcinoma	HSC3/4	migration, MMP	[55]
	Breast cancer	MCF-7/MDA-MB-231	migration	[56]
	Lewis Lung cancer	LLC-2	adhesion	[33]
	Glioblastoma	U-87MG/T98G	migration/chemotaxis	[57]
	Pancreatic cancer	Panc-1	migration, invasion	[58]
	Prostate cancer	PC-3 LNCaP	migration cell survival	[59] [60]
TRPV2	Prostate cancer	PC-3	migration	[61]
	Breast cancer	MCF-7/MDA-MB-231	migration	[62]
TRPV4	Breast cancer	MDA-MB-435s *	migration, invasion, metastasis, transendothelial migration	[26,63]
	Gastric cancer	MKN45 and SGC-7901	proliferation, migration	[64]
TRPV6	Breast cancer	MCF-7/MDA-MB-231/tissue	migration/chemotaxis expression in invasive areas	[65]
	Pancreatic cancer	Pancreatic cancer cells/tissue	proliferation, migration, invasion	[66]

* Recently recognized as of melanoma origin [67].

**Table 2:** TRP channel expression in different cancer types and its correlation with patient prognosis.

Channel	Cancer Type	Expression	Prognosis	Ref.
TRPC1	Breast cancer Basal tumors/lymph nodes	high	poor	[25]
TRPC5	Colon cancer	high	poor	[42]
TRPC6	Glioblastoma	high	no information	[43]
	Prostate cancer	high	no information	[45]
	Esophageal squamous cell carcinoma	high	poor	[68]
TRPM2	Breast cancer	low	poor	[27]
TRPM7	Pancreatic cancer PDAC/lymph nodes	high	poor	[28,47,69]
	Breast cancer Negative (ER(-)) invasive ductal carcinoma/lymph nodes	high high	poor poor	[49] [50,51]
TRPM8	Urothelial carcinoma of bladder	high	poor	[29]
	Osteosarcoma	high	poor	[30]
TRPV2	Prostate cancer	high	poor	[61]
	Breast cancer	high	better	[31]
	Esophageal squamous cell carcinoma	high	poor	[32]
TRPV4	Breast cancer	high	poor	[26,63]
	Gastric cancer	high	poor	[63]
	Ovarian cancer	high	poor	[63]
TRPV6	Breast cancer	high	poor	[65]
	Pancreatic cancer	high	no information	[66]
